# Open questions on the reactivity of Criegee intermediates

**DOI:** 10.1038/s42004-021-00483-5

**Published:** 2021-03-25

**Authors:** Rebecca L. Caravan, Michael F. Vansco, Marsha I. Lester

**Affiliations:** 1 Chemical Sciences and Engineering Division, Argonne National Laboratory, Lemont, IL USA; 2 Department of Chemistry, University of Pennsylvania, Philadelphia, PA USA

**Keywords:** Reaction kinetics and dynamics, Atmospheric chemistry

## Abstract

Criegee intermediates are reactive intermediates formed in Earth’s atmosphere through ozonolysis of alkenes. Here the authors outline the fundamental chemistry that influences their highly conformer- and substituent-dependent unimolecular and bimolecular reactivity, and discuss open questions of fundamental and atmospheric interest.

Criegee intermediates (CIs) are carbonyl oxide reactive intermediates with zwitterionic character that are formed from the ozonolysis of unsaturated hydrocarbons. Until recently^1^, direct generation and detection of these short-lived intermediates had not been realized, and thus our understanding of CI reactivity was based on careful analysis of complex steady state chamber studies. It was these experiments that first identified the potentially important role that CIs could play in Earth’s lower atmosphere, e.g., the formation of sulfate aerosols in the troposphere from alkene ozonolysis in the presence of SO_2_^2,3^. Recent alternative methods for generation of CIs using diiodo-alkane and -alkene precursors facilitated direct detection, characterization, and kinetic studies of CIs in the laboratory^1,4^. These new studies revealed the structural and conformational dependence of CI reactivity, highlighting the importance of direct experimental and high-level theoretical studies to complement steady-state chamber investigations representing complete reaction sequences.

Significant efforts have been made to understand the reactivity of the three isoprene-derived CIs (formaldehyde oxide, CH_2_OO; methyl vinyl ketone oxide, MVK-oxide; and methacrolein oxide, MACR-oxide; see Fig. 1 for chemical structures) motivated by both atmospheric pertinence and fundamental chemical interest. Isoprene is the most abundant non-methane hydrocarbon emitted into the atmosphere (ca. 600 Tg/year)^5^. It is a five-carbon, conjugated diene, which is released into Earth’s troposphere by trees and plants. Ozonolysis is an important sink of tropospheric isoprene (~10%) and results in the production of both one- and four-carbon CIs^6^. The vast predicted and observed differences in their unimolecular and bimolecular reactivity, despite their common origin and, for a subset of these, minimal structural differences, make these species particularly interesting from a fundamental standpoint^7–9^. Their remarkably different reactivity is illustrated in Fig. 1 utilizing known or predicted rate constants and concentrations for the primary atmospheric reactants (water vapor, SO_2_, and organic acids) from the Amazon region^1,7,8,10–14^. In this Comment, we discuss recent findings from direct studies of these isoprene-derived CIs, focusing on the four-carbon unsaturated CIs. We present some open, fundamental questions for future CI studies, and consider how these studies could contribute to understanding of real-world phenomena.

**Fig. 1:**
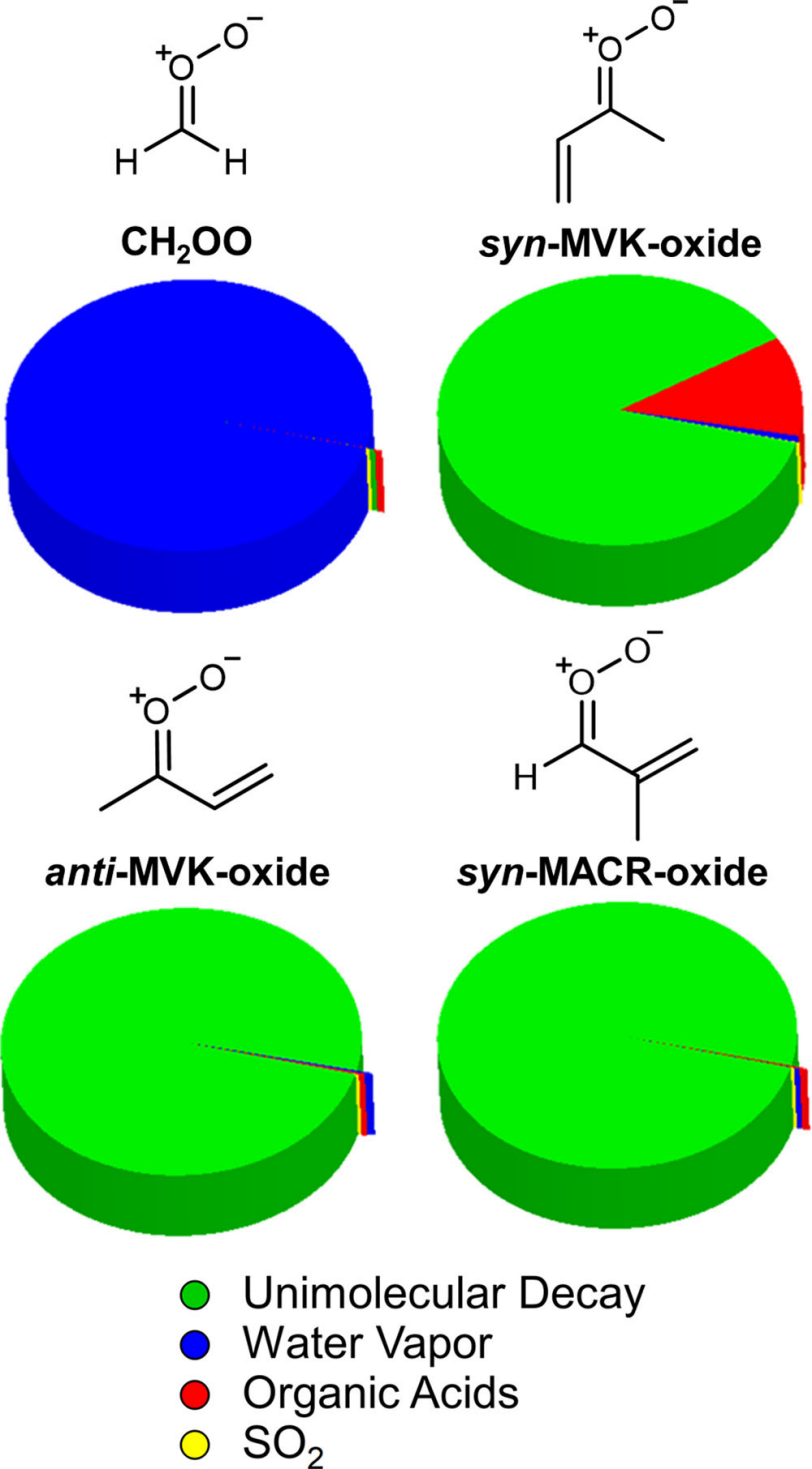
Pie charts illustrating relative contributions of major atmospheric reaction pathways. The contributions of major unimolecular and bimolecular reactions for isoprene-derived Criegee intermediates (CH_2_OO, MVK-oxide, and MACR-oxide) under tropospheric conditions predicted for the Amazon region are shown^13,14^. Experimental studies of *anti*-MACR-oxide reactions are an emerging focus^19^ and thus not included here.

## Reactivity of the simplest Criegee intermediate

**Fig. 2:**
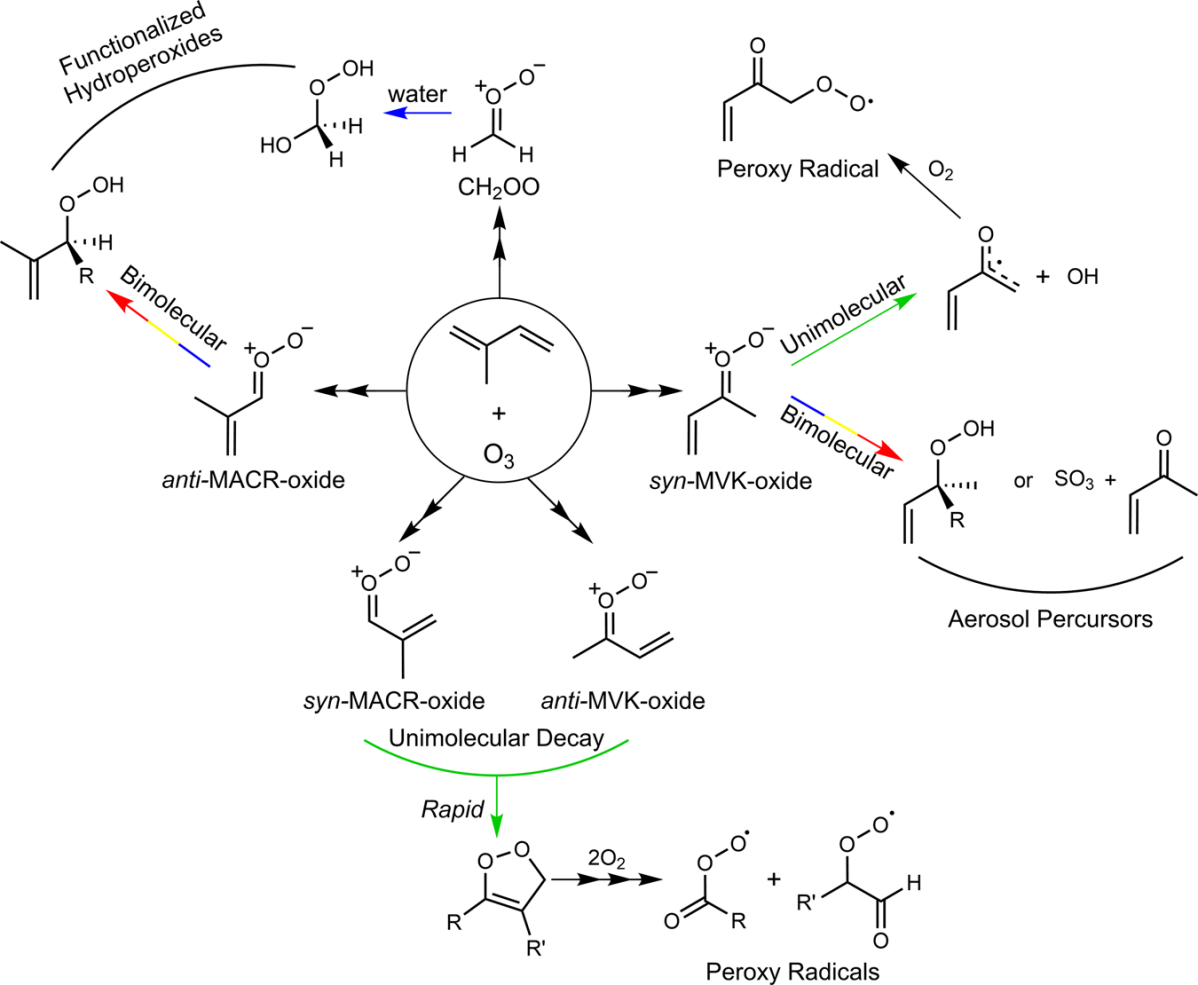
Schematic illustration of major atmospheric reaction pathways. Primary unimolecular processes and bimolecular reactions for isoprene-derived Criegee intermediates (CH_2_OO, MVK-oxide, and MACR-oxide) in the atmosphere are shown. Many of the pathways lead to reactive hydroxyl (OH) or peroxy radicals, while others involve addition reactions that generate functionalized hydroperoxides and aerosol precursors. R represents various substituent groups.

CH_2_OO is formed with a 58% yield in isoprene ozonolysis^6^. CH_2_OO has been the subject of numerous experimental and theoretical studies since 2012^1^, when it was directly generated with sufficiently high yields to facilitate direct spectroscopic, kinetic and mechanistic characterization. The experimental work revealed that CH_2_OO reacts with SO_2_ at a rate 10,000 times faster than anticipated based on analysis of chamber studies^1^. Further studies demonstrated rapid reaction with organic acids—leading to functionalized hydroperoxide species via a 1,4-addition mechanism^11^. By contrast, unimolecular decay of CH_2_OO and its bimolecular reaction with water monomers were found to be slow^7^. However, subsequent studies at higher water concentrations found the reaction of CH_2_OO with water dimers to be extremely rapid—indeed, so fast that it is predicted to dominate the removal of CH_2_OO from the atmosphere^15,16^. Theoretical and experimental studies have revealed that this reaction leads to the formation of hydroxymethyl hydroperoxide (HMHP)—a functionalized hydroperoxide species also formed from reaction of CH_2_OO with water monomers (see Fig. 2)^10^.

## Unimolecular decomposition of four-carbon unsaturated Criegee intermediates

The four-carbon unsaturated CIs, MVK-oxide and MACR-oxide, are generated with yields of 23 and 19%, respectively, in isoprene ozonolysis^6^. MVK-oxide and MACR-oxide are isomers, both having vinyl and methyl substituents, but differing in the position of the methyl groups. Both have extended conjugation across the vinyl and carbonyl oxide groups that fundamentally changes their electronic structure and impacts their unimolecular and bimolecular chemistry in the atmosphere^8,12,17^. MVK-oxide and MACR-oxide each have four conformational forms with similar ground state energies (within ca. 3 kcal mol^-1^); the four conformers fall into two groups, separated by high barriers (∼30 kcal mol^-1^ for MVK-oxide^8^), which are distinguished by the orientation of the terminal oxygen with respect to the vinyl group (*syn* and *anti*)^8,17^. Under atmospheric conditions, the two conformers within each group (*cis* and *trans*) rapidly interconvert by rotation about the C-C bond^12^. Quite amazingly, the distinct conformational forms of MVK-oxide and MACR-oxide undergo remarkably different unimolecular decay processes with rates that differ by orders of magnitude^7,8^.

The *syn* conformers of MVK-oxide undergo slow thermal unimolecular decay (33 s^−1^) to OH radical products (Fig. 2)^8^. The relatively slow decay rate compared to simple alkyl-substituted CIs is attributed to the loss of extended conjugation and the resultant higher transition state barrier for unimolecular decay. By contrast, thermalized *anti*-conformers of MVK-oxide and analogous structural conformers of MACR-oxide undergo a unique decay mechanism with extremely rapid (2140 s^−1^ and 2500 s^−1^, respectively) ring closure to form dioxoles, 5-membered cyclic peroxides, which are formed with sufficient internal excitation to rapidly rearrange and release oxygenated hydrocarbon radical products (Fig. 2)^7,18^. In the atmosphere, these radicals rapidly react with O_2_ to form peroxy radicals that quickly decay to stable carbonyl products.

## Bimolecular reactions of four-carbon unsaturated Criegee intermediates

The slowly decaying conformational forms of the four-carbon unsaturated CIs, MVK-oxide (*syn*) and MACR-oxide (*anti*), can undergo bimolecular reactions with atmospherically abundant water vapor, SO_2_, and organic acids. Bimolecular encounters of MVK-oxide and MACR-oxide with water vapor (monomers and dimers) cause disruption of the extended conjugation in these CIs, and result in reaction barriers that are substantially higher than those for CH_2_OO^7,12^. This effect, along with steric hindrance arising from the substituents, dramatically reduces the predicted—and recently observed—rate coefficients for the reactions of the four-carbon unsaturated CIs with water vapor compared to CH_2_OO^12,19^. As a result, bimolecular reaction with water vapor is not expected to be the dominant atmospheric loss process for MVK-oxide or MACR-oxide.

By contrast, the rates coefficients for reaction of MVK-oxide with SO_2_ and formic acid are as large as those for CH_2_OO^12^. For MVK-oxide, theoretical study of its bimolecular reactions indicate that the barriers are comparatively higher than CH_2_OO, but strongly submerged relative to reactants, such that bimolecular reaction is facile with SO_2_ and formic acid. Similar reaction profiles are anticipated for MACR-oxide, where rapid reaction with SO_2_ is also observed^19^.

The rapid bimolecular reactions of specific conformers of MVK-oxide and MACR-oxide with SO_2_ and organic acids indicates that these reactions could play important roles in the troposphere. Global modeling indicates the reaction of MVK-oxide with SO_2_ contributes to sulfuric acid production, ultimately generating sulfate aerosols, while reaction of MVK-oxide with formic acid leads to its significant removal over the Amazon. Analogous global modeling for the bimolecular loss pathways of MACR-oxide is a future challenge.

## Open questions and outlook

We anticipate that ozonolysis of many biogenic alkenes will yield more complex functionalized CIs, and that their unimolecular decay rates to OH radical products may be strongly impacted. Theoretical calculations and derived structure-function relationships predict new types of H-atom migration processes for unsaturated CIs: allylic 1,4 H-atom shift and allylic 1,6 H-atom shift reactions^7^. These processes are predicted to significantly enhance H-migration rates and enable H-atom migration over longer ranges, thereby increasing the rates of unimolecular decay to OH products.

In addition, CIs with heteroatom substituents are fundamentally interesting and relatively unexplored. These CIs are important in atmospheric ozonolysis of endocyclic alkenes, such as cyclic terpenes and terpenoids with high biogenic emissions^5^. The ring opening associated with ozonolysis will form bifunctional CIs with carbonyl oxide and carbonyl groups, which may have a significant impact on their unimolecular and bimolecular reactivity. Moreover, new low energy pathways leading to rapid intramolecular secondary ozonide (SOZ) formation are predicted to become efficient for sufficiently large CIs^20^. The latter is particularly relevant for secondary organic aerosol (SOA) formation.

The role of CIs in gas-particle interconversion remains an area of significant interest. The pathways by which CIs can drive the formation of higher molecular weight, lower volatility SOA precursors, e.g., via the formation of functionalized hydroperoxides, requires further exploration. This is of particular importance for more complex and functionalized CIs including MVK-oxide and MACR-oxide, and for bimolecular reactions where functionalized hydroperoxides are formed as reaction products. For example, this occurs in reactions of CIs with water vapor, amines, alcohols, and organic acids.

There are numerous examples in the literature showing that the rate and branching fraction of gas-phase bimolecular reactions are influenced by the presence of water vapor. The influence of single water molecule complexation on the reactivity of CIs has recently been experimentally explored for the first time^21^. Developing a comprehensive understanding of how such complexation impacts reaction rates and product branching fractions of unimolecular and bimolecular reactions of reactive intermediates, such as the CIs formed from isoprene ozonolysis, is of significant fundamental interest and atmospheric pertinence. Furthermore, such studies will connect our current understanding of the role and reactivity of CIs in the gas phase to heterogenous environments.
